# Early pathogenesis profiles across SARS-CoV-2 variants in K18-hACE2 mice revealed differential triggers of lung damages

**DOI:** 10.3389/fimmu.2022.950666

**Published:** 2022-10-27

**Authors:** Zhen Qin Aw, Chee Keng Mok, Yi Hao Wong, Huixin Chen, Tze Minn Mak, Raymond T. P. Lin, David Chien Lye, Kai Sen Tan, Justin Jang Hann Chu

**Affiliations:** ^1^ Biosafety Level 3 Core Facility, Yong Loo Lin School of Medicine, National University of Singapore, Singapore, Singapore; ^2^ Department of Microbiology and Immunology, Yong Loo Lin School of Medicine, National University of Singapore, Singapore, Singapore; ^3^ Infectious Disease Translational Research Programme, Yong Loo Lin School of Medicine, National University of Singapore, Singapore, Singapore; ^4^ National Public Health Laboratory, National Centre for Infectious Diseases, Singapore, Singapore; ^5^ Infectious Disease Research and Training Office, National Centre for Infectious Diseases, Singapore, Singapore; ^6^ Department of Infectious Diseases, Tan Tock Seng Hospital, Singapore, Singapore; ^7^ Department of Medicine, Yong Loo Lin School of Medicine, National University of Singapore, Singapore, Singapore; ^8^ Lee Kong Chian School of Medicine, Nanyang Technological University, Singapore, Singapore; ^9^ Department of Otolaryngology, Yong Loo Lin School of Medicine, National University of Singapore, Singapore, Singapore; ^10^ Collaborative and Translation Unit for Hand, Foot and Mouth Disease (HFMD), Institute of Molecular and Cell Biology, Agency for Science, Technology and Research, Singapore, Singapore

**Keywords:** SARS-CoV-2, variants of concern, immune response, cytokine storm, K18-hACE2 mice model

## Abstract

The on-going COVID-19 pandemic has given rise to SARS-CoV-2 clades and variants with differing levels of symptoms and severity. To this end, we aim to systematically elucidate the changes in the pathogenesis as SARS-CoV-2 evolved from ancestral to the recent Omicron VOC, on their mechanisms (e.g. cytokine storm) resulting in tissue damage, using the established K18-hACE2 murine model. We reported that among the SARS-CoV-2 viruses tested, infection profiles were initially similar between viruses from early clades but started to differ greatly starting from VOC Delta, where the trend continues in Omicron. VOCs Delta and Omicron both accumulated a significant number of mutations, and when compared to VOCs Alpha, Beta, and earlier predecessors, showed reduced neurotropism and less apparent gene expression in cytokine storm associated pathways. They were shown to leverage on other pathways to cause tissue damage (or lack of in the case of Omicron). Our study highlighted the importance of elucidating the response profiles of individual SARS-CoV-2 iterations, as their propensity of severe infection *via* pathways like cytokine storm changes as more variant evolves. This will then affect the overall threat assessment of each variant as well as the use of immunomodulatory treatments as management of severe infections of each variant.

## Introduction

The emergence of severe acute respiratory syndrome coronavirus 2 (SARS-CoV-2) has resulted in the coronavirus disease 2019 (COVID-19) pandemic that caused major healthcare and economic burden worldwide (1). The infection SARS-CoV-2 caused is highly heterogeneous ranging from asymptomatic and mild to severe and even death (2). In addition, the heterogeneity is further enhanced by the rapid mutation of the virus giving rise to different clades and variants that caused varying pathologies over the course of the pandemic (3). Hence, it is imperative to characterize the differential pathology of SARS-CoV-2 clades and variants and their mechanisms to improve management of the infection, especially for development of therapies that are based on the modulation of these mechanisms.

Coronaviruses have been shown to recombine extensively due to their proofreading mechanism that can facilitate homologous recombination events, giving rise to novel variants of varying pathology and severity (4). To date, SARS-CoV-2 is clustered into 9 major clades based on the Global Initiative on Sharing All Influenza Data (GISAID) database (5, 6). Clade L is the first novel viral genome sequence serving as the reference ancestral strain (7), and other representative clades with major genetic mutations include clades S, V, G, GK, GH, GR, GV, and GRY. Among the clades, clade G and its derivatives have rapidly become the dominant globally circulating SARS-CoV-2 that gave rise to the major variants of concern (VOCs) (8), including Alpha (B.1.1.7), Beta (B.1.351), Gamma (P.1), Delta (B.1.617.2) and Omicron (B.1.1.529). The VOCs have caused concerns as they were shown to have increased virulence, greater transmissibility, resistance to antibody neutralization, and evasion to host- and vaccine-mediated immunity (9–11). It is understood that the SARS-CoV-2, especially the VOCs, resulted in different degrees of severity and symptoms in humans and animal models (12, 13). However, due to the rapid evolution of each VOCs and them rarely overlapping in emergence, there have yet to be a systematic comparison of the early triggers of airway and lung damage among the major SARS-CoV-2 variants, especially between all VOCs.

Several studies have shown that the expression of pro-inflammatory cytokines is associated with disease progression in COVID-19 patients (14–17). Hypercytokinemia, also known as cytokine storm, is one of the major triggers for tissue damage, multiple organ dysfunction, and lethal outcomes in SARS-CoV-2 infection (18, 19). Further, it is well documented that the build-up of activated neutrophils and macrophages in the airways and lungs following SARS-CoV-2 infection results in increased pro-inflammatory cytokines that contribute to airway pathology (20). On the other hand, the evolution of SARS-CoV-2 VOCs over the course of the pandemic showed great variation in their severity and pathology, which is especially apparent in Omicron VOC, suggesting differential cytokine activation that contributes to their pathogenesis (21). Henceforth, we employed the established K18-hACE2 mouse model to systematically compare the disease phenotypes across clades and variants and correlate them with their respective early response cytokine profiles, to determine involvement of cytokine storm and selected pathways’ contribution to their pathogenesis. We aim to elucidate the evolutionary progression of SARS-CoV-2 pathogenesis to understand the pathology spectrum of the virus, which will help in current and future management and drug discovery effort against the virus.

## Materials and methods

### Cell line and SARS-CoV-2 variants

African green monkey kidney cells, (Vero E6; ATCC CRL-1586™) were cultured in Dulbecco’s Modified Eagle’s Medium (DMEM; Cytiva) supplemented with 10% heat-inactivated fetal calf serum (FCS).

SARS-CoV-2 viruses were isolated from nasopharyngeal swabs of qRT-PCR confirmed COVID-19 patients and propagated in Vero E6 for infection experiments, where low passages of not more than 4 passages of the virus were used. Genome sequences from the swab samples uploaded to GISAID by the National Public Health Laboratory, National Centre for Infectious Diseases, Singapore, were used to confirm the variants’ identity. A total of eight viruses were used: 1. Clade L (ancestral), Lineage B (EPI_ISL_574502); 2. Clade V, Lineage B.29 (EPI_ISL_493419); 3. Clade G, Lineage B.1.610 (EPI_ISL_443240); 4. Clade GR, Lineage B.1.1 (EPI_ISL_443199); 5. VOC Alpha variant, Clade GRY (thereafter GRY-α), Lineage B.1.1.7, (EPI_ISL_754083); 6. VOC Beta variant, Clade GH/501Y.V2 (thereafter as GH-β), Lineage B.1.351.3 (EPI_ISL_1173248); 7. VOC Delta variant, Clade GK (thereafter GK-δ), Lineage B.1.617.2 (EPI_ISL_2621925); 8. VOC Omicron variant, Clade GRA (thereafter GRA-o), Lineage B.1.1.529 (EPI_ISL_7195620). All virus experiments were performed in NUS Medicine biosafety level 3 (BSL-3) core facility and all protocols were approved by the BSL-3 biosafety committee (BBC) and the institutional biosafety committee (IBC) of the National University of Singapore (NUS).

### Experimental infection

Eight to nine weeks-old female K18-hACE2 transgenic mice (InVivos Ptd Ltd, Singapore) were acclimatized in the ABSL-3 facility for 72 hours prior to infection and were infected with approximately 1 × 10^3^ PFU of SARS-CoV-2 virus suspension in PBS, *via* the intranasal route. Baseline body weight were measured prior to virus infection. Body weight, physiological conditions, and survival were monitored daily by two personnel for the duration of the experiment (14-days post infection, dpi) or until the humane endpoint was reached. Each infection group was performed at n=6, except variant GRY-α at n=5, and mock infection at n=3. Scoring of the infected mice physiological conditions were based on five criteria: appearance of mouse coat, level of consciousness, activity level, eye condition, and respiratory quality. Conditions were scored on a scale from 1 to 5, using an observation system adapted from Shrum, et al. (22), with 1 denoting normal physiological state and 5 being the most severe. Additional groups of mice at n=7, except GK-δ at n=5, and GRA-o at n=6 were sacrificed on 4 dpi to assess the viral load in the brain, lung, liver, and spleen; and histological analyses of the lung. Tissues were halved and homogenized in DMEM supplemented with antibiotic-antimycotic and the supernatant was used for plaque assay, while the remaining tissue pellets were kept in -80°C for RNA isolation. The other half of the tissue was fixed with 3.7% formaldehyde (10% formalin) solution for at least 19 h before removal from BSL-3 containment for histological analysis. All animal experiments were conducted in NUS Medicine Biosafety Level 3 (ABSL-3) facility in accordance with NUS IACUC protocol no. R20-0504, using NUS IBC and BBC approved SOPs.

### Plaque assay

For virus titre determination, viral supernatants from homogenized tissues were serially diluted in 10-fold increments in DMEM supplemented with antibiotic-antimycotic (Gibco). 250 µl of each serially diluted supernatant were added to confluent Vero E6 cells and incubated for 1 h at 37°C with rocking at 15 min intervals. After 1 h of adsorption, virus inoculum was removed, and cells were washed once with phosphate-buffered saline (PBS). Overlay media containing 1.2% microcrystalline cellulose-DMEM supplemented with antibiotic-antimycotic were added to each well and incubated for 3 days at 37°C at 5% CO_2_ for plaque formation. Cells were fixed in 10% formalin overnight and stained with crystal violet for plaque visualization. Number of plaques were determined, and virus titre of individual samples were expressed in logarithm of plaque forming units (PFU) per organ.

### Histopathological analyses

Formaldehyde fixed tissues were routinely processed, embedded in paraffin blocks (Leica Surgipath Paraplast), sectioned at 5 µm thickness, and stained with hematoxylin and eosin (H&E; Thermo Scientific) following standard histological procedures. A multiparametric, semiquantitative scoring system was further used to assess the magnitude of histo-morphological and -pathological changes in lung tissues based on six criteria: inflammatory cell infiltrates, hemorrhage, edema, degeneration of alveolar epithelial cells, parenchymal wall expansion and bronchiole epithelial cell damage (23, 24). For the histopathological parameters, a score of 0 – 3 was ordinally assigned, where 0 indicated normal; 1 indicated less than 10%; 2 indicated 10 – 50%; and 3 indicated more than 50% of lung regions affected. The average of histopathological scores of the mice from each group was taken as the final evaluation index.

### Immunohistochemistry (IHC)

The paraffin embedded lung sections were deparaffinized and rehydrated, followed by heat-mediated antigen retrieval process. The sections were then treated with hydrogen peroxide blocking and protein blocking for 10 min, respectively. After blocking the non-specific binding sites, sections were incubated with anti-SARS-CoV-2 nucleocapsid protein monoclonal antibody (1:1000; Abcam), followed by the respective horseradish peroxidase (HRP)-conjugated secondary antibody. The sections were subsequently visualized using DAB solution and counterstained with hematoxylin.

### RNA isolation and qRT-PCR

Homogenized lung tissue pellets were subjected to total RNA extraction using QIAGEN RNeasy^®^ mini kit in accordance with manufacturer’s guidelines. RNA purity and concentration were determined using Tecan Infinite^®^ M200 Pro coupled with NanoQuant Plate™. Real-time reverse transcription polymerase chain reaction (qRT-PCR) was performed using QIAGEN RT^2^ First Strand Kit, QIAGEN RT^2^ SYBR^®^ Green Mastermix, and QIAGEN RT^2^ Profiler PCR Array (PAMM-150ZE-4) in accordance with manufacturer’s recommendations.

### Gene expression analysis

Murine cytokines mRNA expression levels from cDNA conversion were analyzed using QIAGEN GeneGlobe Design and Analysis hub, and gene expression were expressed as Log_2_ fold regulations. Expression levels were calculated relative to *Gapdh* and normalized to the mock infected mice using the ΔΔC_T_ method with the QIAGEN GeneGlobe software. The genes from the QIAGEN panel were further sub-divided into selected pathways that may potentially contribute to tissue damage using DAVID functional annotation tool (25, 26). Pathways were selected from Gene Ontology (GO) and Kyoto Encyclopedia of Genes and Genomes (KEGG) databases for visualization of enrichment in each pathway across variants.

### Statistical analysis

Analyses were calculated using two-tailed Mann-Whitney U test to evaluate the data obtained using Graphpad Prism version 9.0 with * denoting that *p* < 0.05, ** denoting that *p* < 0.01, and *** denoting *p* < 0.001. For gene expression analysis, p-values were obtained from the analysis with QIAGEN GeneGlobe Design and analysis hub. Only genes fulfilling both criteria of >2 fold-regulation compared to uninfected controls and having a p-value of <0.05 were considered significant.

## Results

**Figure 1:**
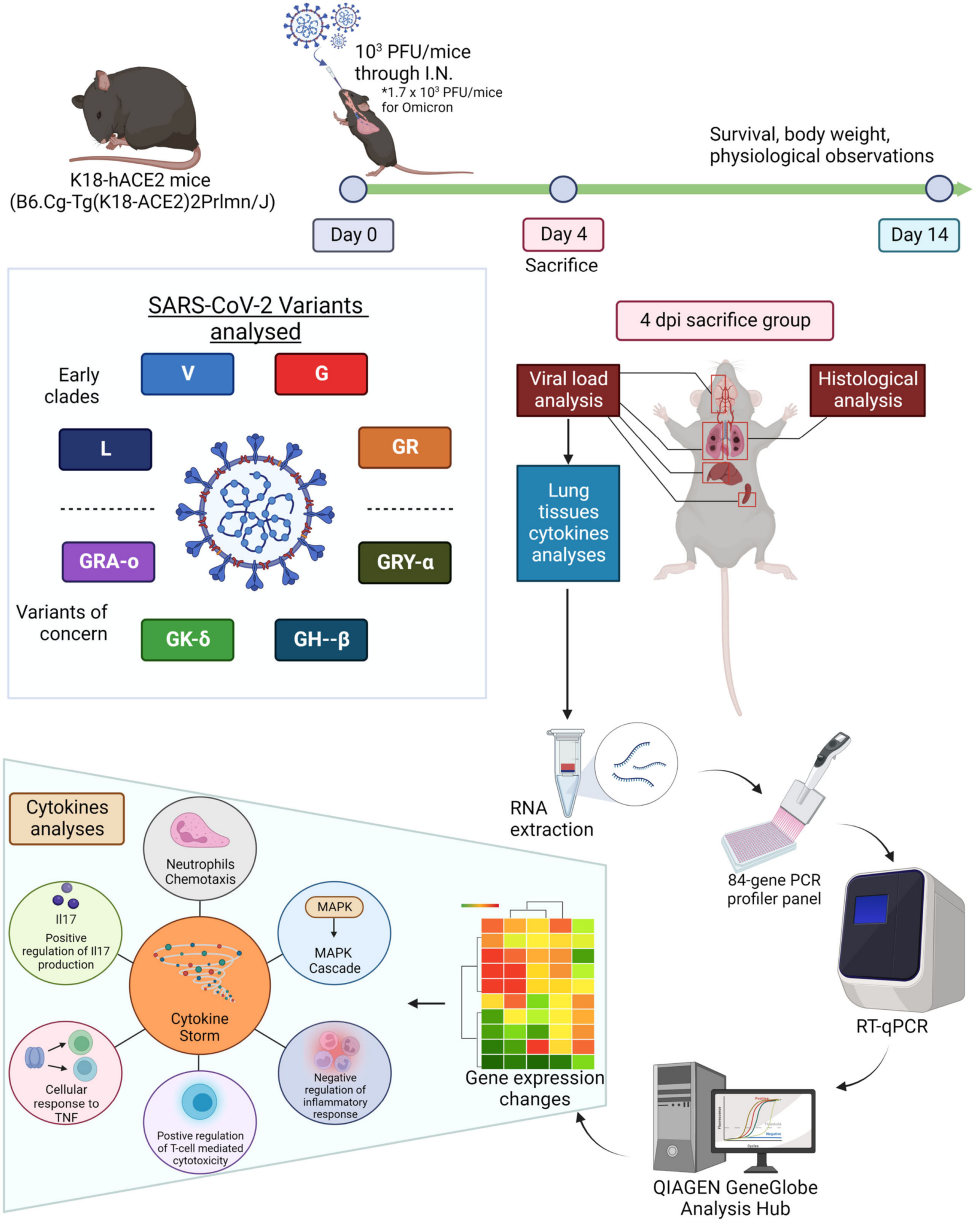
Summarized methodology of characterization of eight viral iterations (ancestral to Omicron) of SARS-CoV-2. K18-hACE2 mice were infected the eight different SARS-CoV-2 viruses and divided into survival group and 4 dpi group (sacrificed 4 days post infection). The survival group were used for assessment of survival and physiological assessment. The 4 dpi group were sacrificed and have organs harvested for virologic, histologic and molecular profiling analysis (cytokine and chemokine gene expressions). Figure created with BioRender.com.

To characterize the pathogenesis of various SARS-CoV-2 viruses from different clades and VOCs, and their respective mechanisms leading to airway and lung damage, we tracked a total of eight major SARS-CoV-2 iterations – L (ancestral), V, G, GR, GRY-α, GH-β, GK-δ, and GRA-o (**Figure 1**). The repertoire of our coverage includes the major SARS-CoV-2 clades and variants of the pandemic to date. The mutations across the iterations based on their clinical isolates’ sequences obtained from GISAID were listed in **Table 1**.

**Table 1:** Amino acid residues signatures of SARS-CoV-2 viruses.

Clade	ORF1ab																																	
	NSP2							NSP3														NSP4		NSP5				NSP6						
	85		188		223		405	38	183	488	837	890	1041	1228	1265	1266	1412	1424	1469	1699	1778	167	492	90	116	132	296	37	77	105	106	107	108	189
**L^1^**	T		I		T		M	K	T	A	K	A	Y	P	S	L	I	S	P	S	N	V	T	K	A	P	V	L	T	L	S	G	F	I
**V**			T															F										F						
**G**																																		
**GR**													H																					
**GRY-α**									I			D					T														∆	∆	∆	
**GH-β**	I										N			L							S			R							∆	∆	∆	
**GK-δ**					I					S				L					S			L	I		V		I		A					
**GRA-ο**								R							Δ	I							I			H				Δ	Δ	Δ		V
Clade	ORF1ab											Spike																						
	NSP12				NSP13				NSP14			S																						
	323		671		77		151		42	157	394	13	18	19	67	69	70	80	95	142	143	144	145	156	157	158	211	212	214	215	222	242	243	244
**L^1^**	P		G		P		I		I	L	A	S	L	T	A	H	V	D	T	G	V	Y	Y	E	F	R	N		Ins	D	A	L	A	L
**V**																																		
**G**	L																																	
**GR**	L																																	
**GRY-α**	L															∆	∆					∆												
**GH-β**	L						V			F		I	F					A												G		∆	∆	∆
**GK-δ**	L		S		L						V			R					I	D				G	∆	∆								
**GRA-ο**	L								V						V	Δ	Δ		I	D	Δ	Δ	Δ				Δ	I	EPE					
Clade	Spike																																	
	S																																	
	339	371	373	375	417	440	446	452	477	478	484	493	496	498	501	505	547	570	583	614	655	679	681	701	716	764	796	856	950	954	969	981	982	1118
**L^1^**	G	S	S	S	K	N	G	L	S	T	E	Q	G	Q	N	Y	T	A	E	D	H	N	P	A	T	N	D	N	D	Q	N	L	S	D
**V**																																		
**G**																				G														
**GR**																				G														
**GRY-α**															Y			D		G			H		I								A	H
**GH-β**					N						K				Y					G				V										
**GK-δ**								R		K										G			R						N					
**GRA-ο**	D	L	P	F	N	K	S		N	K	A	R	S	R	Y	H	K			G	Y	K	H			K	Y	K		H	K	F		
Clade	ORF3a								Envelope		Membrane				ORF7a		ORF7b	ORF8			Nucleocapsih													
	NS3								E		M				NS7a		NS7b	NS8			N													
	16		26		57	171	175	251	9	71	3	19	63	182	82	120	40	27	52	73	3	13	31	32	33	63	203	204	205	214	215	220	235	377
**L^1^**	K		S		Q	S	T	G	T	P	D	Q	A	I	V	T	T	Q	R	Y	D	P	E	R	S	D	R	G	T	G	G	A	S	D
**V**								V																										
**G**																																		
**GR**																											K	R						
**GRY-α**							I	V										X	I	C	L						K	R					F	F
**GH-β**					H	L				L																			I					
**GK-δ**			L											T	A	I	I									G	M			∆	∆			Y
**GRA-ο**									I		G	E	T									L	Δ	Δ	Δ		K	R						

Comparison of amino acid substitutions with each SARS-CoV-2 viruses’ open reading frame arising from mutations in the genomic sequence.

^1^Based on CoVSurver comparison to GISAID reference strain hCoV-19/Wuhan/WIV04/2019. (Reference: GISAID - CoV surver mutations app [Internet].) [Retrieved April 11, 2022]. Available from: https://www.gisaid.org/epiflu-applications/covsurver-mutations-app/.

### Changes in survival, symptoms, and tissue tropism over the course of SARS-CoV-2 evolutionary iterations

**Figure 2:**
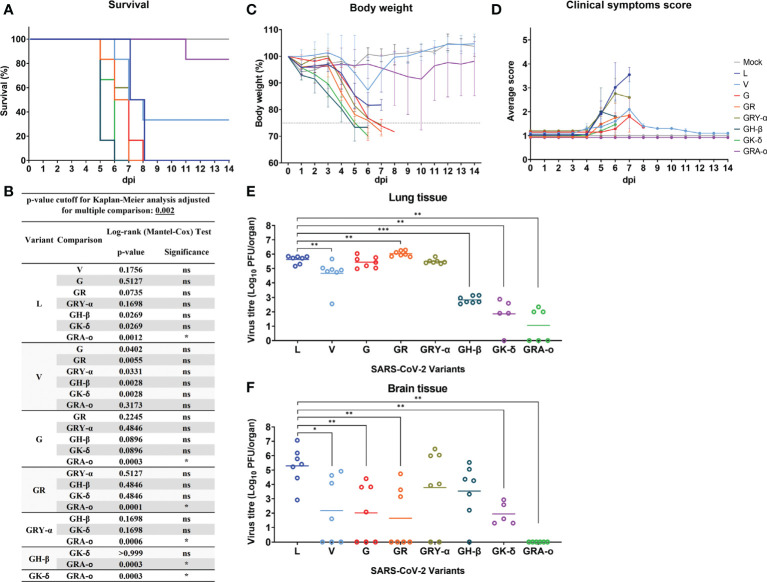
SARS-CoV-2 viruses infected K18-hACE2 transgenic mice. Physiological conditions and weight changes of infected mice group across different SARS-CoV-2 viruses (L, V, G, GR, GRY-α, GH-β, GK-δ, and GRA-o), and uninfected mice over 14 dpi. Mice were infected with approximately 10^3^ PFU of the respective SARS-2-CoV viruses through intranasal delivery. **(A)** Survival percentage of infected mice over 14 days, **(B)** Significance comparison of survival curves for SARS-CoV-2 infected mice. Comparison of significance between survival curves of various variant infected mice using log-rank (Mantel-Cox) test. Cut off for p-value using Kaplan-Meier analysis was adjusted to 0.002 for multiple comparison between various survival curves. **(C)** percentage changes in body weight, **(D)** changes in physiological condition quantified through scoring of clinical symptoms. Individual mouse was scored across 14 days from 1 (normal) to 5 (worst) for five conditions: coat appearance, level of consciousness, activity, eye condition, and respiration. To determine clinical score of the infected mice, average of the five physiological parameters for each infection group. All infection groups, n=6 with except of variant G, n=5, and mock infected mice, n=3. Error bars denotes range for each time point. **(E, F)** For viral load comparison after infection, mice were sacrificed at 4 dpi (L, V, G, GR, GRY-α, GH-β: *n = 7*; GK-δ: *n = 5*; GRA-o: *n = 6*) with lungs, brain, liver, and spleen tissues harvested for titration. SARS-CoV-2 virus titre in lung and brain tissue determined *via* plaque assays. Geometric mean of viral titre is shown. Statistical significance between clade L and variants determined with two-tailed Mann-Whitney U test. *p < 0.05, **p < 0.01 and ***p < 0.001.

We first compared the survival of K18-hACE2 mice following infection with the different SARS-CoV-2 viruses. It was observed that, with the exception of V (34% survival) and GRA-o (83.3% survival), 0% of infected mice survived the infection, where they succumbed between 6 to 8 dpi, with GH-β and GK-δ hitting the end point the earliest at 6 dpi (**Figure 2A**). When comparing the Kaplan-Meier survival curves, it was shown that only GRA-o, the latest evolutionary iteration, was significantly different in mice survival compared with all other infected groups except for V, another iteration of the virus where there were mice that survived (**Figure 2B**). The weight loss trends largely followed the survival curves in which rapid weight loss were observed between 4 and 7 dpi for all groups other than V and GRA-o, for which we observed a rebound in weight from surviving mice (**Figure 2C**). We then compared clinical symptom scores (averaged from appearance, eye condition, consciousness, activity, and respiration) and observed that L and GRY-α showed the most symptom presentation, followed by the others that showed moderate symptoms, while GRA-o was shown to be not presenting any clinical symptom throughout the duration of study (**Figure 2D**, **Figures S1A–E**). We then examined viral titre retrieved from the tissues (lung, brain, liver, and spleen) of SARS-CoV-2 infected mice. No infectious viruses were recovered from liver and spleen homogenates using plaque assay (data not shown). For viral titre in the lungs, high titres of SARS-CoV-2 was retrieved from the lungs of L, V, G, GR and GRY-α infected mice, at geometric mean titres of 4.27 × 10^5^, 4.55 × 10^4^, 2.75 × 10^5^, 1.09 × 10^6^, and 3.12 × 10^5^ PFU/organ, respectively. Titres from V are slightly lower, while GR slightly higher when compared with L infected group (p < 0.05). On the other hand, significantly lower titre was observed in GH-β, GK-δ and GRA-o when compared with L, at 6.86 × 10^2^, 7.2 × 10^1^ and 1.1 × 10^1^ PFU/organ, respectively (p < 0.05) (**Figure 2E**). As for viral titre in the brain, comparatively high titre was found in the brain tissue of L, GRY-α and GH-β infected groups, at 1.93 × 10^5^, 6.02 × 10^3^ and 3.4 × 10^3^ PFU/organ, respectively; while significantly lower titre in the brain was retrieved in brain tissue of G, GR and GK-δ at 1.03 × 10^2^, 4.4 × 10^1^, and 8.7 × 10^1^ PFU/organ, respectively. No virus was retrieved from the brain of GRA-o infected mice (**Figure 2F**). Notably, unlike in the lung, viral titre in the brain was more variable where about half of the mice did not have viral titre retrieved from the brain in most groups, regardless of the titre observed.

### Early histopathological damage of SARS-CoV-2 viruses were not clearly distinguishable

**Figure 3:**
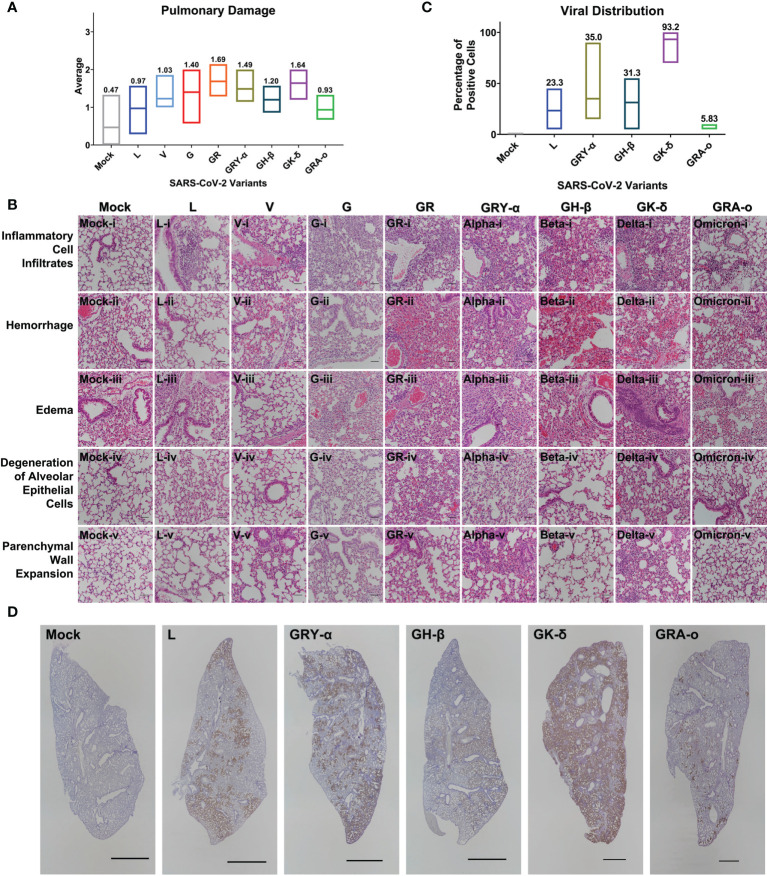
Histopathological and immunohistochemical analyses of lung sections from K18-hACE2 mice following infection with SARS-CoV-2 at 4 dpi. **(A)** Histopathological scoring of lung damage by evaluating the extent and areas of pulmonary involvement based on 6 criteria: inflammatory cell infiltrates, hemorrhage, edema, degeneration of alveolar epithelial cells, parenchymal wall expansion and bronchiole epithelial cell damage, assigned respectively with a score of 0 – 3. The average of the 6 parameters from mice for each group was regarded as the final histopathological scoring. Mice following mock infection are representative of n=3 per group while mice infected with SARS-CoV-2 are representative of n=7 per group (n=5 for GK-δ, and n=6 for GRA-o). **(B)** Hematoxylin and eosin staining of lung sections from mock- or SARS-CoV-2-infected K18-hACE2 mice. H&E-stained lung sections demonstrated massive inflammatory cell infiltrates, hemorrhage and thickened interalveolar septa in the mice following SARS-CoV-2 inoculation. Hemorrhage was observed in the mock-infected mice could be due to a crushing artifact and damaged capillary walls during tissue handling at necropsy. 50x total magnification, scale bars = 50µm. **(C)** Percentage of SARS-CoV-2 nucleocapsid protein positivity in the FFPE lung tissues infected with L, GRY-α, GH-β, GK-δ and GRA-o. **(D)** Detection of SARS-CoV-2 nucleocapsid protein (brown staining) showed the virus was predominantly found in alveolar septa and pneumocytes, but not bronchiole regions. DAB chromogen and hematoxylin counterstain were used. Each image is representative of n=7 per group (L, V, G, GR, GRY-α, GH-β), n=5 (GK-δ), n=6 (GRA-o), n=3 (mock). 5x total magnification, scale bars = 2000µm.

From the lung tissue collected at 4 dpi, it was observed that histopathological damage of SARS-CoV-2 variants was largely similar, with histopathological index score ranging between 0.63 to 1.69. Tissues from GR and GK-δ infected mice were on the higher end of the scores while those from L and GRA-o were with lower scores (**Figure 3A**). The factors that likely contributed to the higher histopathological index were inflammatory cell infiltrates, where the trend was consistent with the overall index compared with other factors (**Figure 3B** **;** **Figures S2A–F**). On the other hand, no histological abnormality was observed in the bronchiole epithelial cells following all variants’ inoculation, and neither bronchitis nor bronchiole epithelial denudation was present in the lung tissues (data not shown). Next, we correlated the extent of infected cells and viral presence in the lungs of the ancestral L infected group, with the VOC infected groups. Using IHC of SARS-CoV-2 nucleocapsid protein, we observed that the viral distribution in the lungs at 4 dpi varied greatly (**Figures 3C, D**). GK-δ, notably, showed major nucleocapsid protein presence in the lung (93.2% coverage), contrary to the low infectious titre detected from the infected murine lungs while GRA-o had almost no nucleocapsid protein detected in the lung tissues (5.83% coverage). Our observation suggested that there was no correlation between nucleocapsid coverage, which are produced in excess during coronavirus infection (27, 28), in the lungs and the lung histopathological damage index between variants.

### SARS-CoV-2 cytokine profiles between evolutionary iterations revealed differences in lung damage triggers

**Figure 4:**
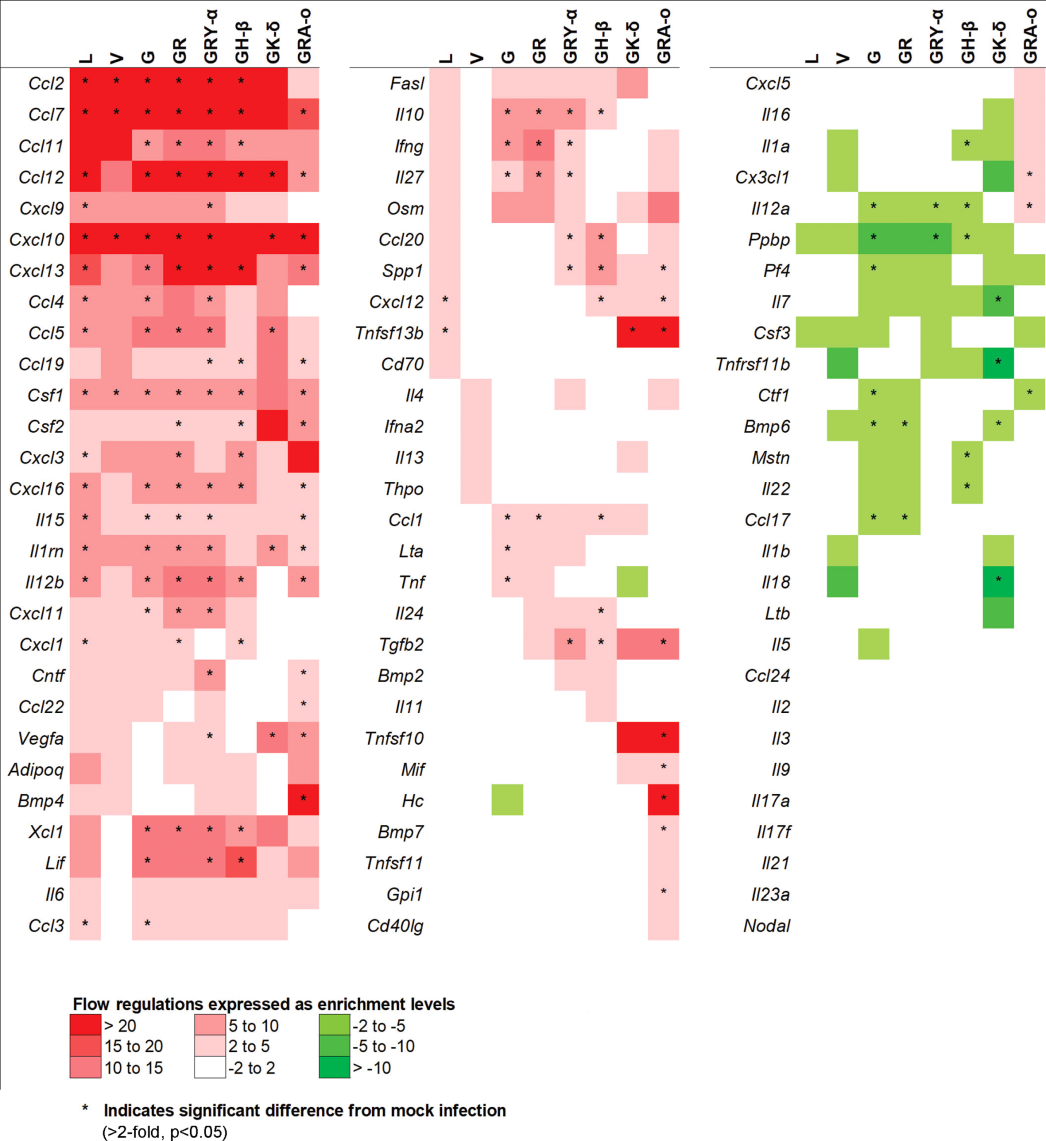
Comparison of 84 cytokines regulation changes among SARS-CoV-2 viruses at 4 dpi. Differentially regulated cytokines across all SARS-CoV-2 virus tested. Color scale indicating level of averaged fold-regulation changes across the groups, * indicate significant difference in fold-regulation changes (>2-fold, p<0.05) between infected and mock infected mice.

To further elucidate potential mechanisms in each of the SARS-CoV-2 viruses that led to their respective phenotypes, we performed a qRT-PCR array on 84 genes related to inflammatory responses from lung tissues of SARS-CoV-2 infected K18-hACE2 mice. After taking into account fold-regulation and statistical significance (>2 fold-regulation and p< 0.05), the cytokine profiles between the infected groups differed, some significantly, from each other. We noted the groups with the larger significant changes from the ancestral L virus (18 up; 0 down) were G (23 up; 6 down), GR (21 up; 2 down), GRY-α (26 up; 2 down), GH-β (21 up; 5 down) and GRA-o (26 up; 1 down). On the other hand, V (4 up; 0 down) and GK-δ (6 up; 4 down) had fewer significant changes compared with L (**Figure 4**). The exact changes in fold-regulation of the genes tested were shown in **Table S1**. In general, the host responses of the SARS-CoV-2 infection of all groups tested deviated significantly from the ancestral L virus, with some having unique significant responses as observed in G (*Lta*, *Tnf*, *Pf4*), GH-β (*Il24*, *Il1a*, *Mstn*, *Il22*), GK-δ (*Il7*, *Tnfsf11b*, *Il18*), and GRA-o (*Ccl22*, *Bmp4*, *Tnfsf10*, *Mif*, *Hc*, *Bmp7*, *Gpi1*, *Cx3cl1*). In view of the differences present, particularly in GRA-o which has the most of them, further dissection on how the differential cytokine changes led to the pathogenesis of current and future variants is thus warranted.

**Figure 5:**
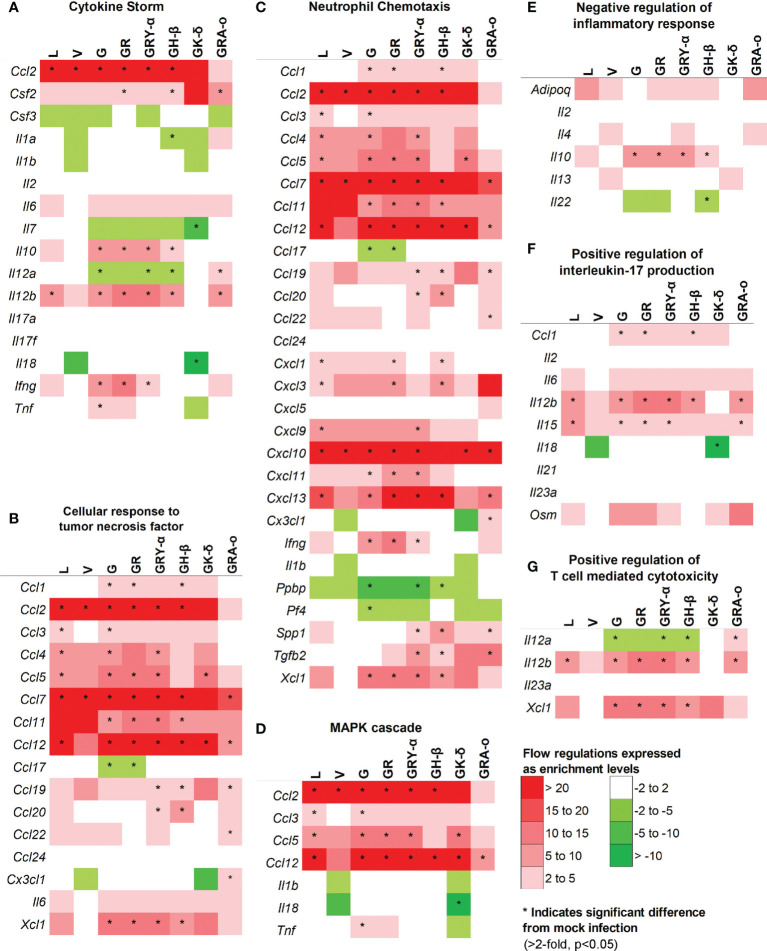
Comparison of differentially expressed genes in selected pathways relevant to cytokine storm and tissue damage at 4 dpi. Differentially regulated cytokines across all groups in pathways related to **(A)** cytokine storm; **(B)** cellular response to TNF **(C)** neutrophil chemotaxis **(D)** MAPK cascade **(E)** negative regulation of inflammatory responses **(F)** positive regulation of IL-17 production **(G)** positive regulation of T-cell mediated cytotoxicity. Color scale indicate level of averaged fold-regulation changes across the groups, * indicate significant difference in fold-regulation changes (>2-fold, p<0.05) between infected and mock infected mice.

As cytokine storm was perceived to be a major trigger of lung damage in COVID-19 (18, 19), we grouped the panel of genes we tested into pathways associated with cytokine storm using DAVID. We selected pathways involved directly in cytokine storm (19), and associated pathways like MAPK cascade, TNF responses, neutrophil chemotaxis, IL-17 regulation, negative regulation of inflammatory response, and T-cell mediated cytotoxicity to determine how much they played a role in tissue damage for each virus iteration (**Figure 5**). Firstly, in the cytokine storm pathway, we observed that for iterations that come after L and V, more genes such as *Ifng*, *Il12b* and *Il10* were found to be significantly differentially expressed in G, GR, GRY-α and GH-β, before being reduced again in GK-δ and GRA-o (**Figure 5A**). Similar trends were observed in TNF response and neutrophil chemotaxis pathways in genes like *Xcl1*, *Ccl1* and *Ccl11* (**Figures 5B, C**). The MAPK cascade pathway was prominently differentially expressed in G, GR, GRY-α and GH-β, but in this case, L and GK-δ as well (**Figure 5D**). On the other hand, negative regulation of inflammatory responses early in infection were linked with cytokine storm (29), and we found that G, GR, GRY-α and GH-β further have differentially up-regulated expression of *Il10* (**Figure 5E**). IL-17 pathway, associated with neutrophil infiltration with genes from the pathway like *Il15* and *Il12b* were up-regulated in all groups except V and GK-δ (**Figure 5F**). Finally, T-cell mediated cytotoxicity pathway was found to be prominently changed for G, GR, GRY-α, GH-β, and GRA-o (**Figure 5G**). Interestingly, only GRA-o had a further significant up-regulation of *Il12a*. Overall, genes from cytokine storm and associated pathways (*Ccl2*, *Ccl7* and *Ccl12*) were found to be differentially regulated in infections of L, G, GR, GRY-α, and GH-β while not as strongly involved in V and GK-δ. GRA-o, while having similar trend of cytokine storm associated pathway gene changes, had more subdued expression, and a slight difference in T-cell mediated cytotoxicity (*Il12a*).

## Discussion

The use of K18-hACE2 murine model to understand SARS-CoV-2 infection has been instrumental to the understanding of SARS-CoV-2 pathogenesis mechanisms since the start of the pandemic, including comparisons of early clades’ differential severity (13, 30–34). Moreover, the K18-hACE2 mice were shown to be simulating severe SARS-CoV-2 infection useful in the identification of immune responses leading to severe outcome (35). Hence, here we reported results using the model to correlate early cytokine profiles (4 dpi) of early SARS-CoV-2 evolutionary iterations (L, V, G, GR), and the VOCs Alpha (GRY-α), Beta (GH-β), Delta (GK-δ) and Omicron (GRA-o), with their infection outcome. Interestingly, the majority of the pathogenesis mechanism and infection outcome remained largely similar up until the Beta VOC, before major changes occur in VOCs Delta and Omicron that emerged later. This may be partially due to the larger number of mutations accumulated (**Table 1**) for Delta and Omicron VOC, which potentially altered their biology significantly.

Our study demonstrated that early cytokine profiles, viral titre and histopathological damage worked in concert to influence and determine the infection outcome. Overall, the infection outcome remained largely similar from L to GH-β Beta VOC, and then deviated in VOCs that emerged late in the pandemic i.e. GK-δ Delta and GRA-o Omicron VOCs. Among the early clades and VOCs up to Beta, even the milder clade V virus with ~30% survival rate had similar rates of neurotropism based on viral titre from brain tissue, sufficiently high viral titre in the lungs, and cytokine profiles consistent with previous report (36). While clade V virus had fewer significantly differentially expressed genes, the major genes involved in cytokine storm; TNF pathway and neutrophil chemotaxis, *Ccl2* and *Ccl7* remained, suggesting similar, but milder presentation of pathology for its infection outcome. The subsequent clade and VOCs in the earlier part of the pandemic, G, GR, GRY-α and GH-β had further inflammatory suppression gene *Il10* differentially up-regulated that potentially augmented their pathology slightly, as seen in the histopathological index. Furthermore, the viruses from earlier in the pandemic, up to VOC GH-β, showed higher clinical presentation scores, which may be linked to neurotropism, prominent in these groups of infections.

On the other hand, VOCs that emerged later in the pandemic, GK-δ Delta and GRA-o Omicron, followed a different pathogenesis mechanism that resulted in tissue damage. Firstly, neurotropism was observed to be less apparent in Delta, and completely absent in the Omicron variant in the K18-hACE2 mice. It was observed that mechanisms that may contribute to cytokine storm were also less apparent in GK-δ and GRA-o infection. In addition, immune suppression, a mechanism common in SARS-CoV-2 (37), was observed strongly in the GK-δ Delta variant, albeit different from *Il10* mediated suppression observed in clades and VOCs that came before. The Delta variant in our study instead showed an almost universal suppression of host responses early post infection at 4dpi (with very little significant differential regulation). This suppression may potentially contribute to the increased nucleocapsid expression and inflammatory infiltrates in the lungs, which may suggest diffusely infected cells in the lungs. On the other hand, the GRA-o variant showed the most varied response among the variants where we observed the least tissue damage, low viral titre and lack of neurotropism. This observation is in line with the finding that Omicron variant has more preference to the upper airway than the lungs (38). In addition, the cytokine profile of our Omicron GRA-o variant infection showed a response largely different than that of its predecessors. It had generally weaker changes in expression of cytokine storm and related pathway genes, coupled with moderately stronger T-cell mediated cytotoxicity gene expression in *Il12a* and *Il12b*. The Omicron cytokine profile suggests a different pathogenesis that may contribute to the overall milder tissue damage congruent with other studies to date (21, 39). The effects of the major changes in Omicron cytokine profiles require further studies to validate their correlation with disease outcome, as well as potential interaction with risk factors and chronic conditions (e.g. asthma) due to their major differences and lower lethality.

The finding from our study is crucial in the understanding of SARS-CoV-2 pathogenesis mechanisms. It showed the evolutionary potential of SARS-CoV-2 where its ability to accumulate high number of mutations may result in highly variable pathogenesis and infection severity in the variants tested and may further change in future variants (4, 40). This is particularly evident in our elucidation of Delta and Omicron cytokine profiles suggesting a major change in the biology of the VOCs late in the pandemic (41, 42). Indeed, the emergence of Delta and Omicron variants both caused major outbreaks that dwarfed those that of their predecessors (43, 44). Studies have shown that Delta and Omicron VOCs has evolved into their distinct evolutionary group and therefore may explain the significant differences of their infection profiles and pathogenesis mechanisms (41). In addition, other reasons such as gestation and adaptation in immunocompromised patients (45), spill-over and spill-back to and back from animal reservoirs (45–47), and potential vaccine induced selection pressure (48), may all contribute to the accumulation of these mutations and the differential pathogenesis mechanisms. Overall, our study inferred that the adaptation of the virus may likely lead to emergence of future variants having mutations that drives their pathogenesis differently than existing SARS-CoV-2 viruses. Therefore, this implied that management strategies based on immunomodulation and mediation of host responses have to be revisited in different variants, mirroring the management of influenza, where propensity of cytokine storm differs (49). Finally, our finding may also partially explain the continued efficacy of broad spectrum immunomodulators like corticosteroids; but not more targeted therapies targeting specific pathways due to the potential differences in different SARS-CoV-2 iterations’ pathogenesis mechanisms.

Our study, however, is not without its limitation. We only elucidated the histopathology and cytokine profile at 4 dpi, that only gave us insights of host response early during infection. This also created potential disconnect such as the case between the high Delta nucleocapsid detection in IHC compared to its low lung infectious viral titre. Nevertheless, high viral protein, especially in the case of nucleocapsid, which were produced in excess during infection (28), may not necessarily translate to infectious viral titre. In addition, we only use female mice for our comparative study and thus may not capture any potential sex specific differences between the cytokine profiles of different virus iterations (50). However, a previous study showed that cytokine and chemokine responses between male and female were largely similar, where the major differences lie at the magnitude and timing of the responses (51). Therefore, our study remained representative of the differences in cytokine profiles between variants despite only testing it in female mice. Nevertheless, future studies with male mice can be performed to investigate the extent of differential cytokine expression levels that the different SARS-CoV-2 virus iteration can induce. Finally, we also did not manage to assess the protein levels of the cytokines in our study. Despite the limitations however, our data showed that, by systematically comparing the profiles across the major evolutionary iterations of SARS-CoV-2, a clear heterogeneity of SARS-CoV-2 evolution and adaptation was observed, especially in VOCs Delta and Omicron that emerged later

In conclusion, our study provided insights on the evolution of pathogenesis and its potential mechanisms of SARS-CoV-2 throughout the course of the pandemic. We have observed that adaptation throughout the pandemic can give rise to variants of very different pathogenesis mechanisms that contribute to tissue damage. Our study thus emphasized the importance in differentiating host responses to different SARS-CoV-2 variants. This is especially true as we enter the endemic phase, where the surveillance of differential host responses between variants will become increasingly crucial when assessing future variants, for their threat and impact, as well as when devising immunomodulatory treatments for severe infections.

## Data availability statement

The original contributions presented in the study are included in the article/**Supplementary Material**. Further inquiries can be directed to the corresponding authors.

## Ethics statement

The animal study was reviewed and approved by NUS Institutional Animal Care and Use Committee (IACUC) under protocol no. R20-0504.

## Author contributions

CKM and JC conceived and designed the experiments. CKM, ZA, TM, DL, and RL contributed to the virus isolation and sequencing. CKM, ZA, HC, and YW performed the experiment. CKM, YW, and KT performed the histological analyses. ZA, CKM, and KT performed the cytokine analyses. CKM, ZA, YW, KT, and JC performed the data analyses. CKM, ZA, YW, KT, and JC wrote the manuscript. All authors have read and approved the final version of the manuscript prior to submission.

## Funding

This research was supported by the following grants: NUHSRO/2020/066/NUSMedCovid/01/BSL3 Covid Research Work, NUHSRO/2020/050/RO5+5/NUHS-COVID/4, Ministry of Education, Singapore MOE2017-T2-2-014, Singapore NMRC Centre Grant Program – Diabetes, Tuberculosis and Neuroscience CGAug16M009, Ministry of Health MOH-COVID19RF2-0001.

## Acknowledgments

We are grateful to the National University of Singapore, Yong Loo Lin School of Medicine BSL-3 Core Facility for their support with this work.

## Conflict of interest

The authors declare that the research was conducted in the absence of any commercial or financial relationships that could be construed as a potential conflict of interest.

## Publisher’s note

All claims expressed in this article are solely those of the authors and do not necessarily represent those of their affiliated organizations, or those of the publisher, the editors and the reviewers. Any product that may be evaluated in this article, or claim that may be made by its manufacturer, is not guaranteed or endorsed by the publisher.

## Supplementary material

Supplementary Table 1 Murine lung mRNA fold regulation changes of 84 selected cytokines and chemokines induced by different SARS-CoV-2 infections. Expression changes in Log_2_ fold-regulation and p-values of 84 mRNA isolated from infected mice lung tissues at 4 dpi separated into L, V, G, GR, GRY-α, GH-β, GK-δ, and GRA-o respectively, when compared to uninfected control.

Supplementary Figure 1 Physiological and symptomatic comparison for SARS-CoV-2 infected mice. Individual physiological condition changes of all eight SARS-CoV-2 infected mice groups over 4 to 6 dpi. Breakdown of individual parameter changes over time from 4 to 6 dpi for **(A)** appearance, **(B)** eye condition, **(C)** level of consciousness, **(D)** activity, and **(E)** respiration respectively.

Supplementary Figure 2 Histopathological comparison of the extent and areas of lung damage inflicted with different SARS-CoV-2 variants. Blinded scoring and quantification of lung injury in respect of **(A)** inflammatory cell infiltrates **(B)** hemorrhage **(C)** pulmonary edema **(D)** degeneration of alveolar epithelial cells, and **(E)** parenchymal wall expansion. Data are presented as average of the histopathological scoring from mice for each group. **(F)** Hematoxylin and eosin-stained images correspond to the mice following mock infection or infection of SARS-CoV-2 (L, GRY-α, GH-β, GK-δ and GRA-o). Images show low-power (top; 5x total magnification; scale bars = 2000µm) and medium-power magnification (bottom; 50x total magnification; scale bars = 50µm). SARS-CoV-2-infected mice are representative of n=7 per group (variants L, V, G, GR, GRY-α, GH-β), n=5 (GK-δ), and n=6 (GRA-o) while mock-infected mice are representative of n=3.
