# Supplementary material for: Early pathogenesis profiles across SARS-CoV-2 variants in K18-hACE2 mice revealed differential triggers of lung damages
Source: Front Immunol. 2022 Oct 27;13:950666. doi: 10.3389/fimmu.2022.950666 (PMC9648130; doi:10.3389/fimmu.2022.950666)
Supplement: Supplementary Table 1 — Murine lung mRNA fold regulation changes of 84 selected cytokines and chemokines induced by different SARS-CoV-2 infections. Expression changes in Log2 fold-regulation and p-values of 84 mRNA isolated from infected mice lung tissues at 4 dpi separated into L, V, G, GR, GRY-α, GH-β, GK-δ, and GRA-o respectively, when compared to uninfected control. [file Table_1.pdf]

Supplementary Table 1.

Variants	*L*		*V*		*G*		*GR*		*GRY-α*		*GH-β*		*GK-δ*		*GRA-ο*	
Gene symbol	**Fold-regulation**	**p-value**	**Fold-regulation**	**p-value**	**Fold-regulation**	**p-value**	**Fold-regulation**	**p-value**	**Fold-regulation**	**p-value**	**Fold-regulation**	**p-value**	**Fold-regulation**	**p-value**	**Fold-regulation**	**p-value**
Adipoq	5.71	0.093209	3.16	0.161327	1.41	0.514358	2.15	0.301117	2.05	0.229401	2.94	0.269808	-1.84	0.171913	5.28	0.333706
Bmp2	1.8	0.494714	-1.32	0.628036	1.31	0.877661	-1.14	0.704741	2.88	0.288647	3.42	0.116416	-1.43	0.44869	1.51	0.751131
Bmp4	4.52	0.251975	2.81	0.619347	1.34	0.515821	1.65	0.635374	3.73	0.297825	2.65	0.757739	1.66	0.423815	23.03	0.001293
Bmp6	-1.12	0.761699	-2.68	0.076861	-3.6	0.000726	-2.87	0.008416	-1.76	0.015759	-1.89	0.00481	-3.61	0.007912	-1.4	0.299634
Bmp7	1.97	0.303543	1.78	0.438875	-1.73	0.226833	1.55	0.513061	1.41	0.73918	1.19	0.973994	1.46	0.466204	3.13	0.045139
Ccl1	-1.04	0.883886	1.28	0.497301	2.23	0.01359	2.85	0.010665	3.05	0.06651	2.61	0.006896	4.46	0.144951	-1.06	0.583169
Ccl11	34.19	0.093281	35.78	0.128983	14.37	0.031581	17.74	0.001667	15.15	0.000001	7.56	0.022276	9.36	0.303669	12	0.06477
Ccl12	31.02	0.034839	15.05	0.061081	21.94	0.00071	34.22	0.000905	23.17	0.037548	23.96	0.006447	124.46	0.002266	11.36	0.009364
Ccl17	1.09	0.581578	-1.05	0.695487	-2.25	0.002623	-2.58	0.007095	-1.13	0.992113	1.08	0.632592	1.08	0.197023	1.11	0.609442
Ccl19	4.52	0.06787	5.36	0.083248	2.93	0.103151	3.59	0.054567	4.99	0.013718	3.77	0.025794	13.89	0.078692	3.97	0.017281
Ccl2	29.17	0.002083	27.71	0.032427	28.53	0.00054	42.4	0.000028	31.65	0.009506	34.23	0.00197	23.42	0.10465	3.3	0.088661
Ccl20	2.17	0.264642	1.21	0.543646	-1.2	0.795045	1.1	0.670467	3.06	0.011512	8.87	0.005424	1.06	0.724128	4.11	0.053359
Ccl22	4.17	0.056901	4.03	0.148091	2.33	0.463108	1.67	0.980229	2.9	0.214958	1.19	0.635809	-1.6	0.900675	4.99	0.022845
Ccl24	-1.31	0.025015	1.06	0.687107	-1.34	0.008687	-1.39	0.016158	-1.17	0.088277	-1.35	0.092715	1.02	0.86863	-1.1	0.323407
Ccl3	3.81	0.035549	1.75	0.493931	3.41	0.013989	3.68	0.075394	2.05	0.235524	2.09	0.284028	4	0.090926	1.26	0.911227
Ccl4	5.82	0.011378	5.28	0.151594	8.78	0.001186	11.48	0.052881	5.32	0.014749	3.45	0.151116	5.3	0.075817	1.2	0.879534
Ccl5	6.5	0.044421	5.8	0.10894	10.06	0.00038	10.74	0.029919	11.16	0.000164	3.71	0.133621	13.8	0.041931	3.85	0.065492
Ccl7	70	0.00285	56.88	0.046148	53.55	0.022705	91.83	0.000021	62.28	0.027516	89	0.001749	160.06	0.06221	17.07	0.017844
Cd40lg	1.38	0.400134	1.72	0.305283	1.08	0.638595	-1.17	0.452099	1.55	0.279938	-1.39	0.26508	1.69	0.480143	2.01	0.261023
Cd70	2.01	0.204381	1.34	0.371583	1.17	0.535072	1.05	0.707074	1.41	0.219346	1.77	0.107329	1.02	0.849906	1.21	0.555978
Cntf	3.07	0.130558	3.96	0.207292	2.43	0.148186	3.54	0.200893	5.44	0.020168	1.63	0.47225	1.76	0.507374	3.8	0.019438
Csf1	8.39	0.048045	5.42	0.021049	5.18	0.037547	9.76	0.003507	9.55	0.005472	9.54	0.000398	12.88	0.065198	5.21	0.000016
Csf2	2.11	0.283128	2.09	0.290495	2.76	0.168011	3.49	0.012312	2.26	0.157922	4.06	0.004321	340.13	0.405814	6.16	0.008886
Csf3	-3.04	0.08798	-3.75	0.060949	-2.98	0.159017	-1.51	0.360569	-2.06	0.106096	-1.41	0.29717	-1.6	0.70128	-3.02	0.059931

Ctf1	-1.28	0.281583	-1.51	0.620376	-2.55	0.037159	-2.44	0.112255	-1.09	0.23025	-1.81	0.037894	-5.56	0.050999	-2.2	0.047028
Cx3cl1	1.15	0.586693	-3.23	0.779111	1.27	0.901832	1.21	0.769441	1.46	0.915173	-1.05	0.460833	-8.12	0.110136	3.54	0.04423
Cxcl1	3.03	0.049242	3.34	0.18342	2.78	0.05386	2	0.001665	1.56	0.195976	3.86	0.016387	-1.12	0.208606	1.07	0.665318
Cxcl10	311.57	0.000015	271.1	0.044389	243.45	0.000435	450.89	0.005016	204.49	0.002679	118.23	0.085052	396.45	0.037884	127.76	0.006398
Cxcl11	4.06	0.197835	4.22	0.210101	3.95	0.003768	6.18	0.003049	6.85	0.002939	2.01	0.200002	1.36	0.4251	1.07	0.619565
Cxcl12	2.44	0.001651	1.87	0.040955	1	0.962285	1.41	0.240502	1.4	0.167598	2.24	0.005951	3.98	0.233792	2.91	0.002597
Cxcl13	17.06	0.047196	6.1	0.103031	13.15	0.038879	22.58	0.001295	30.33	0.002556	45.96	0.000874	6.98	0.111509	11.29	0.011017
Cxcl16	5.24	0.031966	3.98	0.059569	5.66	0.00387	7.49	0.002397	7.01	0.001514	5.76	0.002675	4.1	0.131428	3.76	0.030281
Cxcl3	4.33	0.041354	7.51	0.179041	7.25	0.107656	6.03	0.003214	2.71	0.267177	5.48	0.003939	2.39	0.306716	27.59	0.116488
Cxcl5	-1.18	0.608181	-1.07	0.791117	-1.34	0.008687	-1.39	0.016158	-1.17	0.088342	-1.36	0.012595	1.44	0.474845	3.43	0.172933
Cxcl9	8.55	0.049785	5.91	0.137587	6.89	0.120305	6.72	0.110261	9.29	0.043886	2.69	0.0951	4.68	0.203818	1.08	0.681205
Fasl	2.92	0.717153	-1.05	0.897834	2.73	0.871344	2.78	0.869621	2.91	0.781455	2.71	0.764273	6.03	0.163112	1.58	0.768866
Gpi1	1.58	0.005544	1.42	0.164229	1.08	0.642418	1.37	0.211442	1.55	0.015612	1.45	0.026051	1.39	0.131707	2.02	0.00535
Hc	1.26	0.646225	-1.03	0.539074	-2.01	0.257041	-1.19	0.561483	1.42	0.892804	1.14	0.725662	-1.02	0.875106	33.41	0.020178
Ifna2	1.3	0.420596	2.75	0.352787	1.42	0.239815	1.05	0.714928	1.31	0.5189	1.06	0.652464	1.02	0.86863	1.03	0.791076
Ifng	3.25	0.278503	1.68	0.396134	9.65	0.027519	12.24	0.02975	4.92	0.032449	1.08	0.805971	1.37	0.520308	3.73	0.1664
Il10	4.01	0.175741	1.96	0.371325	8.03	0.028478	9.02	0.011872	6.35	0.009509	4.69	0.048112	1.9	0.479396	1.31	0.474022
Il11	1.73	0.375553	1.43	0.519747	1.39	0.478454	1.45	0.476708	1.53	0.369548	3.08	0.082785	-1.22	0.365744	-1.05	0.741823
Il12a	-1.94	0.444517	-1.34	0.836223	-4.52	0.024382	-2.28	0.182734	-4.76	0.023563	-4.27	0.021623	-1.72	0.587351	3.34	0.001628
Il12b	7.01	0.003542	2.63	0.204048	8.03	0.001976	11.15	0.006959	10.32	0.000024	6.08	0.048981	1.83	0.36719	5.79	0.027769
Il13	-1.27	0.040519	2.18	0.30864	-1.34	0.008687	-1.24	0.208642	-1.17	0.088342	-1.4	0.004164	3.22	0.280352	1.53	0.164533
Il15	5.29	0.010493	3.92	0.106502	3.19	0.018369	3.49	0.005247	3.76	0.000386	2.44	0.080788	3.26	0.188651	2.75	0.01242
Il16	1.52	0.829876	-1.91	0.306777	1.12	0.468814	1.55	0.97912	-1.14	0.269611	-1.88	0.145815	-2.13	0.461981	3.54	0.114552
Il17a	-1.24	0.067228	-1.07	0.791117	-1.34	0.008687	-1.17	0.676985	-1.17	0.088342	-1.25	0.418481	1.02	0.86863	-1.1	0.323407
Il17f	-1.31	0.025015	-1.07	0.791117	-1.34	0.008687	-1.39	0.016158	-1.17	0.088342	-1.4	0.004035	1.46	0.436974	1.53	0.292776
Il18	-1.4	0.256486	-6.98	0.09135	-1.22	0.471125	-1.29	0.398985	-1.01	0.778716	-1.92	0.022197	-21.3	0.046204	-1.06	0.658382
Il1a	-1.18	0.372165	-2.22	0.753416	1.2	0.890574	-1.32	0.347472	-1.21	0.284537	-3.08	0.017779	-3.13	0.191468	3.32	0.062474
Il1b	1.18	0.457795	-2.37	0.798689	1.02	0.855982	1.15	0.562997	-1.33	0.126336	-1.62	0.177747	-2.2	0.168684	1.05	0.660027
Il1rn	5.21	0.000176	5.11	0.108083	6.51	0.000827	7.54	0.001516	6.88	0.019767	2.89	0.128866	5.17	0.034687	3.29	0.005692
Il2	-1.03	0.831322	1.03	0.770307	-1.34	0.008687	-1.39	0.016158	1.01	0.805309	-1.32	0.241224	1.02	0.86863	-1.1	0.323407
Il21	-1.31	0.025015	1.01	0.84144	-1.34	0.008687	-1.39	0.016158	-1.17	0.088342	-1.4	0.004035	1.02	0.86863	-1.1	0.323407

Il22	-1.66	0.292623	1.34	0.55387	-2.07	0.067395	-2.11	0.144954	-1.68	0.154197	-2.23	0.037378	-1.56	0.189586	-1.66	0.118377
Il23a	1.05	0.636288	-1.35	0.168239	-1.7	0.005657	-1.52	0.054294	1.05	0.720906	-1.58	0.016223	-1.24	0.154994	-1.04	0.884667
Il24	1.29	0.437576	1.18	0.52149	1.56	0.411408	2.56	0.150649	3.28	0.154627	3.75	0.040472	1.54	0.251435	-1.1	0.323407
Il27	2.38	0.186956	1.03	0.567012	4.09	0.004579	6.32	0.00303	3.7	0.044	1.79	0.323897	-1.56	0.85234	2.19	0.146026
Il3	-1.03	0.83915	-1.07	0.791117	-1.34	0.008687	-1.39	0.016158	-1.17	0.088342	-1.4	0.004035	1.02	0.86863	-1.1	0.323407
Il4	1.78	0.344272	2.26	0.4454	-1.02	0.727162	1.51	0.425516	2.14	0.058833	1.74	0.186242	1.02	0.86863	2.57	0.255512
Il5	-1.45	0.460059	-1.67	0.266789	-2.02	0.187288	-1.62	0.361932	-1.86	0.175378	-1.92	0.158788	-1.79	0.22969	-1.14	0.504657
Il6	3.35	0.164471	1.66	0.370592	2.05	0.247727	2.09	0.336971	2.27	0.175624	2.17	0.21314	2.18	0.390689	3.24	0.116644
Il7	-1.19	0.460357	-1.93	0.675801	-2.93	0.089609	-2.32	0.155618	-2.2	0.124929	-2.87	0.059023	-7.25	0.038322	-1.47	0.233374
Il9	-1.31	0.025015	-1.07	0.791117	-1.34	0.008687	-1.39	0.016158	-1.17	0.088342	-1.4	0.004035	1.02	0.86863	-1.1	0.323407
Lif	8.65	0.087274	1.33	0.491976	12.98	0.00766	11.59	0.066433	14.08	0.005187	15.76	0.028093	3.6	0.396514	8.25	0.060292
Lta	-1.25	0.786143	-1.02	0.906832	2.85	0.046368	4.8	0.053902	2.69	0.209526	1.58	0.481304	-1.31	0.538025	1.05	0.992893
Ltb	-1.48	0.756512	-1.94	0.656808	1.4	0.43699	1.99	0.136247	1.38	0.443357	-1.04	0.891219	-5.79	0.679378	1.99	0.229661
Mif	1.31	0.583079	1.68	0.356814	1.02	0.727295	1.02	0.783307	1.04	0.805741	1.46	0.280248	4.05	0.098675	2.04	0.039103
Mstn	-1.26	0.815299	-1.77	0.177274	-2.05	0.090845	-2.29	0.152654	-1.94	0.095689	-2.32	0.045653	-1.62	0.193637	-1.82	0.111666
Nodal	-1.24	0.071827	-1.07	0.791117	-1.34	0.008687	-1.39	0.016158	-1.17	0.088342	-1.4	0.004035	1.02	0.86863	-1.1	0.323407
Osm	2.16	0.220161	-1.48	0.839807	5.41	0.056104	6.26	0.06171	2.36	0.148934	1.9	0.458292	3.03	0.23898	11.75	0.057938
Pf4	-1.45	0.376318	-1.92	0.142157	-4.44	0.026513	-2.79	0.117838	-3.02	0.057716	-1.08	0.36109	-2.64	0.121928	-2.05	0.096046
Ppbp	-2.02	0.180506	-4.07	0.060789	-8.04	0.017484	-6.4	0.063364	-7.86	0.018662	-3.46	0.018145	-2.85	0.07522	-1.11	0.212546
Spp1	2.38	0.139021	1.14	0.454216	1.89	0.116341	1.55	0.193935	3.01	0.020079	9.73	0.013218	3.44	0.137253	4.51	0.014123
Tgfb2	1.88	0.405815	1.89	0.374289	1.71	0.532795	2.75	0.123164	5.61	0.003036	4.43	0.027994	11.6	0.127795	13.69	0.000581
Thpo	1.11	0.583306	2.34	0.455823	-1.32	0.010494	-1.16	0.48156	1.19	0.41829	-1.31	0.062952	1.02	0.86863	1.75	0.241565
Tnf	1.7	0.185426	1.51	0.325581	4.47	0.011179	2.8	0.07732	1.68	0.167945	1.75	0.248784	-2.46	0.690884	1.35	0.439073
Tnfrsf11b	1.33	0.286888	-5.01	0.396085	-1.78	0.554519	-1.35	0.29624	-2	0.081156	-2.33	0.080409	-11.91	0.000086	-1.85	0.054808
Tnfsf10	-1.23	0.281659	-1.07	0.791117	-1.34	0.008687	-1.25	0.262272	-1.17	0.088342	-1.4	0.004035	76.19	0.056742	301.79	0.000435
Tnfsf11	-1.1	0.877494	-1.07	0.791117	-1.17	0.259885	-1.15	0.717033	-1.01	0.85937	-1.21	0.875341	1.19	0.329362	2.19	0.08901
Tnfsf13b	3.13	0.008552	-1.03	0.976182	-1.24	0.556985	1.39	0.562758	-1.1	0.569466	-1.73	0.091308	120.12	0.023865	77.8	0.000291
Vegfa	2.72	0.156651	2.09	0.227767	1.35	0.47274	2.24	0.130076	2.54	0.007093	1.7	0.165271	11.35	0.043658	8.85	0.000777
Xcl1	6.86	0.054421	1.99	0.435383	14.72	0.024655	14.8	0.013458	12.13	0.023874	8.64	0.034171	13.81	0.112113	4.47	0.161433
