# Supplementary figures and images for: Early pathogenesis profiles across SARS-CoV-2 variants in K18-hACE2 mice revealed differential triggers of lung damages
Source: Front Immunol. 2022 Oct 27;13:950666. doi: 10.3389/fimmu.2022.950666 (PMC9648130; doi:10.3389/fimmu.2022.950666)

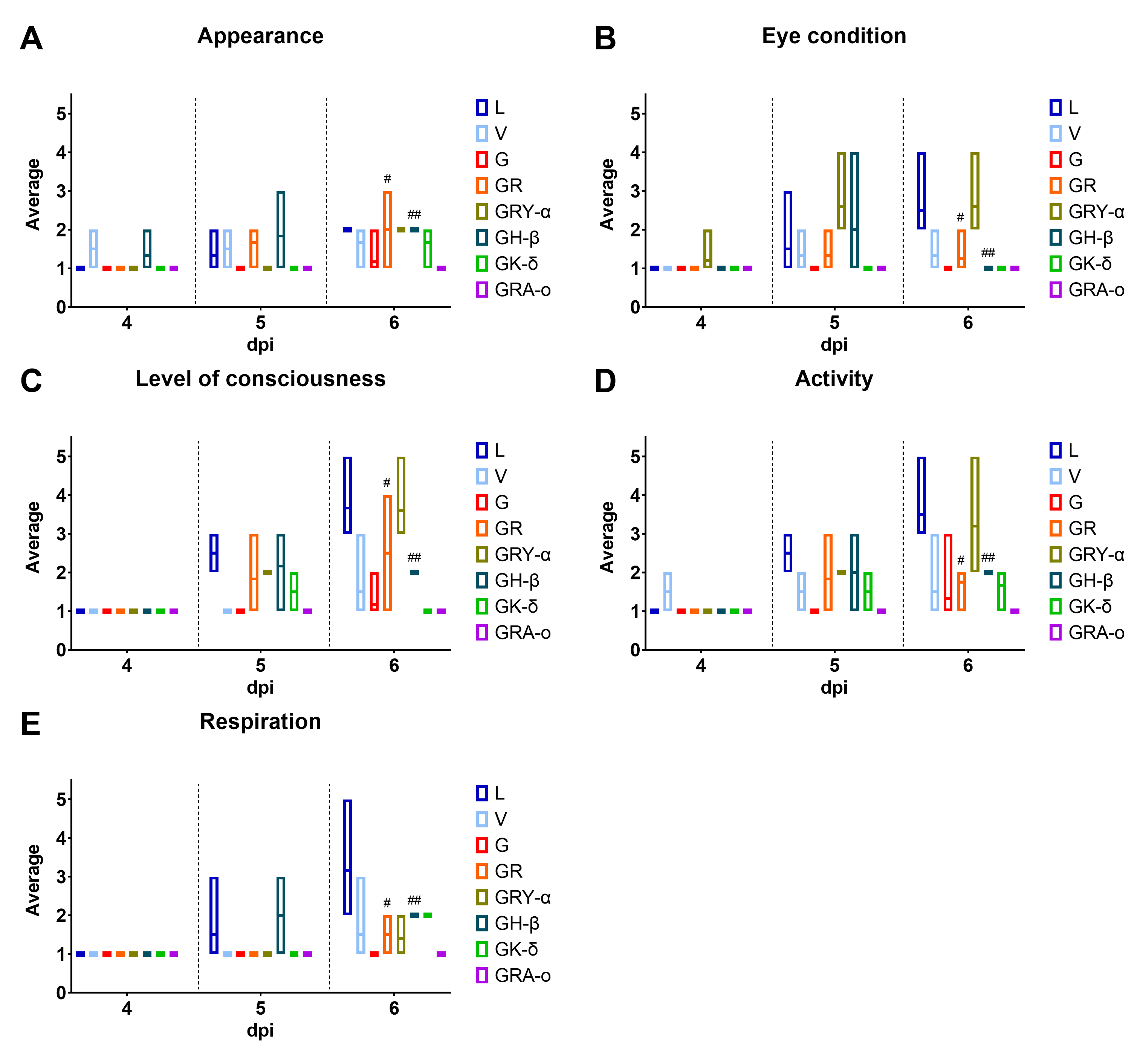

Supplement: Supplementary Figure 1 — Physiological and symptomatic comparison for SARS-CoV-2 infected mice. Individual physiological condition changes of all eight SARS-CoV-2 infected mice groups over 4 to 6 dpi. Breakdown of individual parameter changes over time from 4 to 6 dpi for (A) appearance, (B) eye condition, (C) level of consciousness, (D) activity, and (E) respiration respectively. [file Image_1.tif]

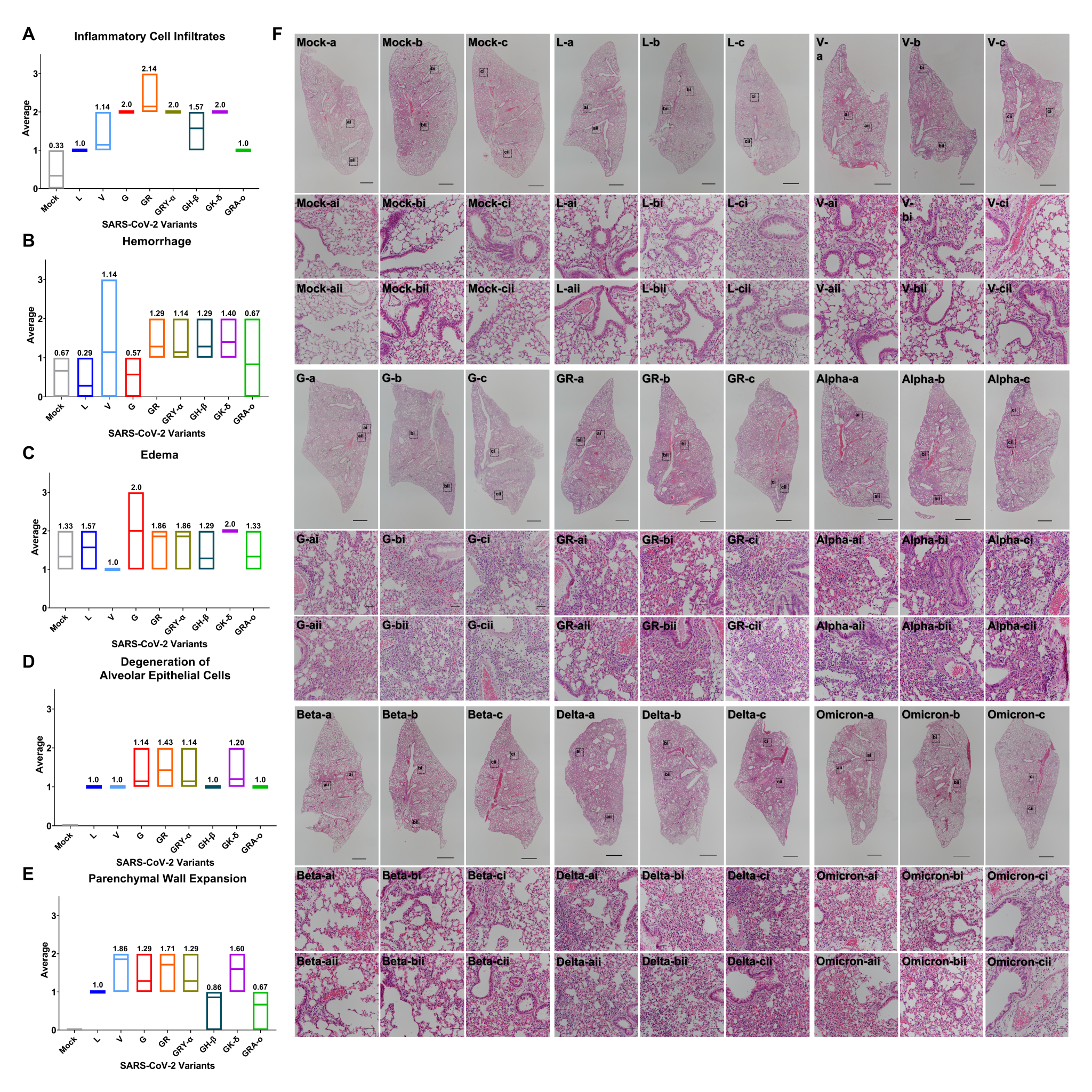

Supplement: Supplementary Figure 2 — Histopathological comparison of the extent and areas of lung damage inflicted with different SARS-CoV-2 variants. Blinded scoring and quantification of lung injury in respect of (A) inflammatory cell infiltrates (B) hemorrhage (C) pulmonary edema (D) degeneration of alveolar epithelial cells, and (E) parenchymal wall expansion. Data are presented as average of the histopathological scoring from mice for each group. (F) Hematoxylin and eosin-stained images correspond to the mice following mock infection or infection of SARS-CoV-2 (L, GRY-α, GH-β, GK-δ and GRA-o). Images show low-power (top; 5x total magnification; scale bars = 2000µm) and medium-power magnification (bottom; 50x total magnification; scale bars = 50µm). SARS-CoV-2-infected mice are representative of n=7 per group (variants L, V, G, GR, GRY-α, GH-β), n=5 (GK-δ), and n=6 (GRA-o) while mock-infected mice are representative of n=3. [file Image_2.tif]
